# Seedless Fruit Production by Hormonal Regulation of Fruit Set

**DOI:** 10.3390/nu1020168

**Published:** 2009-11-23

**Authors:** Tiziana Pandolfini

**Affiliations:** Dipartimento di Scienze, Tecnologie, e Mercati della Vite e del Vino, University of Verona, 37029 San Floriano (Verona), Italy; Email: tiziana.pandolfini@univr.it

**Keywords:** phytohormones, seedless fruit, parthenocarpy

## Abstract

Seed and fruit development are intimately related processes controlled by internal signals and environmental cues. The absence of seeds is usually appreciated by consumers and producers because it increases fruit quality and fruit shelf-life. One method to produce seedless fruit is to develop plants able to produce fruits independently from pollination and fertilization of the ovules. The onset of fruit growth is under the control of phytohormones. Recent genomic studies have greatly contributed to elucidate the role of phytohormones in regulating fruit initiation, providing at the same time genetic methods for introducing seedlessness in horticultural plants.

## 1. Introduction

The biological function of the fruit is the protection of embryos and seeds during their development and the facilitation of seed dispersal after maturation. The onset of fruit development from the ovary, the so-called fruit set, occurs after fertilization of the ovules and it is coordinated by signals produced by the developing embryos. The processes of seed and fruit development which are intimately connected and synchronized, are controlled by phytohormones [1]. Fruit growth can be uncoupled from fertilization and seed development as indicated by the existence of seedless mutant plants (e.g., tomato *pat* mutants) and seedless crops obtained by traditional breeding methods (*i.e.* seedless grape, citrus, cucumber and watermelon) [2,3]. A plant is considered to be seedless when its fruits are completely devoid of seeds, contain a much-reduced number of seeds or present aborted seeds. Seedless fruit can be obtained through parthenocarpy if the fruits develop without fertilization and by stenospermocarpy if the seeds abort after fertilization [3]. In the latter case, the fruits contain traces of the aborted seeds.

Seedlessness is appreciated by consumers both in fruits for fresh consumption (e.g., grape, citrus, and banana) as well as in conserved or processed fruits (e.g., frozen eggplant, tomato sauce). Seedlessness can contribute to increase the quality of the fruits when seeds are hard or have a bad taste. In the case of eggplant, the absence of seeds prevents browning and texture reduction of the pulp [4]. Furthermore, seeds can produce substances that accelerate the deterioration of the fruit, as in watermelon and eggplant. In this regard, the absence of seeds can increase the shelf life of the fruits, allowing a better conservation. 

The independence of fruit development from fertilization is advantageous in horticulture when fruit set rate is low. Pollen maturation and fertilization are affected by environmental factors such as light, temperature, relative humidity. Unfavourable environmental conditions can drastically depress fertilization and consequently fruit development. Parthencarpic plants can circumvent this problem allowing seedless fruit development also under environmental conditions adverse for pollination and/or fertilization. In horticulture, parthenocarpy can be exploited for increasing winter and early production [5,6]; this means the possibility for the consumers to find fresh horticultural products in all seasons.

A widespread agriculture practice for the production of seedless parthenocarpic fruit consists in treating flowers with phytohormones before pollination. Auxin, gibberellin and cytokinins or mixtures of these hormones have all been proven to be effective in inducing fruit development in the absence of fertilization in several crop species, for instance tomato and eggplants [1].

In the last years, the process of fruit development has been the object of many studies aimed to investigate the biochemical and genetic factors that control fruit growth and maturation. More recently, several studies have addressed the role of phytohormones in fruit set regulation at the transcriptomic level demonstrating that many phytohormones concur to regulate this process in a complex way [7,8,9,10]. The majority of these investigations have been carried out on tomato which is considered the model plant for fleshy fruit development. 

This review will describe recent findings that have contributed important knowledge about the role of plant hormones in fruit set, focusing on genetic methods for obtaining seedless fruits by manipulating hormones’ action. 

## 2. Auxin

The phytohormone auxin regulates almost all developmental processes in plants, including fruit development. Natural and artificial auxins supplied exogenously to unpollinated flowers induce fruit growth in tomato and in other horticultural plants, suggesting that these hormones can replace the signals provided by pollination and fertilization [11,12]. This hypothesis is in accordance with the finding that increased auxin levels are detected in flower organs after fertilization of the ovules [1]. 

Recently, molecular analyses have confirmed the prominent role played by auxin signaling in triggering and coordinating the transition from flower to fruit [7,8,13]. The data published so far support the idea that the growth of the ovary is blocked before pollination and that auxin is involved in derepression of ovary growth after fertilization [14].

Auxin is synthesised in meristems and young leaves and transported in a polar fashion to the other parts of the plant [15]. Auxin signaling is mediated by two families of transcription factors: Aux/IAA and ARF. Auxin signal is perceived by F-box receptor proteins and, upon binding of auxin to the receptor, AUX/IAA repressor proteins are ubiquitinated and degraded *via* proteasome. AUX/IAAs and ARFs interact and form complexes that repress auxin signaling and action; the degradation of AUX/IAA proteins allows ARF-mediated regulation of auxin responsive genes [15,16]. In pollination-dependent and pollination-independent fruit set several AUX/IAA and ARF genes display shift in their expression in the transition from flower to fruit, whereas auxin biosynthetic genes are not modulated at the onset of fruit growth [7,8,9]. In accordance with the observations obtained from transcriptomic analyses it has been demonstrated that is possible to uncouple fruit and seed development by manipulating auxin action at different levels affecting auxin biosynthesis, auxin signaling pathway and auxin action (Table 1).

Seedless fruits were obtained by expressing the auxin synthesising gene *iaaM* of *Pseudomonas syringae* pv. *savastanoi* under the control of the ovule/placenta specific promoter from the *DefH9* gene of *Antirrhinum majus* [17,18]. The promoter is active in ovules and placenta and in the tissue derived from there, consequently auxin is synthesised during the whole growth of the fruit [6]. This gene was used to confer parthenocarpy to several horticultural crops including tomato, eggplant, cucumber, raspberry [17,18,19,20]. The *DefH9-iaaM* parthenocarpic tomatoes and eggplants showed an enhanced productivity under environmental conditions unfavourable for pollination associated to a high quality of the fruits [5,6]. The parthenocarpic fruits had the same shape and equal or bigger size as compared with seeded fruits [6,18]. The *DefH9-iaaM* fruits did not contain seeds when grown under conditions adverse for pollination and showed a much reduced number of seeds under optimal environmental conditions [6,21]. The quality of *DefH9-iaaM* tomato fruits is equivalent to that of seeded fruits except for higher beta-carotene content in the parthenocarpic fruit [21]. From a nutritional point of view the increased beta-carotene content can be a valuable trait of *DefH9-iaaM* seedless tomato fruits.

Two components of auxin signalling pathway have been identified as negative regulators of fruit set in tomato. The first one is the *IAA9* gene of tomato coding for a transcription factor belonging to the IAA/AUX family. Downregulation of *IAA9* through an antisense approach resulted in parthenocarpic development of the fruit together with auxin-related alterations in the leaves [22]. The second one is a potential AUX/IAA-interacting protein. Two members of ARF family, *AtARF8* of *Arabidopsis thaliana* and *ARF7* of tomato, have recently been implicated in fruit initiation. Both genes show high level of expression in non pollinated flowers and are downregulated after pollination. *A. thaliana* *ARF8* mutants display parthencarpic silique and the introduction of the Atarf8-4 mutant allele in tomato resulted in the production of seedless fruits [23,24]. A similar phenotype was observed in tomato plants when *ARF7* function was suppressed using RNA interference (RNAi) [25]. Based on these data different models of fruit set control have been postulated. One model suggests that in the ovary, at pre-anthesis, specific AUX/IAA regulatory proteins would form heterodimers with auxin response factors (ARFs) repressing auxin signaling and consequently ovary growth [14]. After successful fertilization of the ovule, a localized burst of auxin causes the degradation via the ubiquitin–proteasome pathway of specific Aux/IAA proteins, releasing ARFs. Free ARFs would subsequently regulate auxin-responsive genes involved in initial phases of fruit growth.

Recently, a new gene family involved in fruit set regulation was identified [26]. The two members of the *AUCSIA* family coding for 53-amino-acid-long peptides are expressed in the ovary and drastically downregulated after pollination. The *AUCSIA* genes were functionally suppressed in tomato by RNAi [26]. *AUCSIA*-silenced plants generally displayed facultative parthenocarpy; these plants produced seedless fruits after flower emasculation. Obligatory parthenocarpy was observed in one *AUCSIA*-silenced line; in this line the fruits were always seedless. Although *AUCSIA* function is unknown, these genes are probably implicated in auxin-regulated processes since *AUCSIA*-silenced plants showed auxin-related phenotypes, an increased auxin level in the flower buds and reduced auxin transport in the roots.

Another genetic approach for the production of seedless fruits is based on the *rolB* gene of *Agrobacterium* *rhizogenes* that alters auxin sensitivity when expressed in plant. The *rolb* gene was introduced in tomato under the control of an ovary and young fruit specific promoter [27]. In the *rolB* transgenic tomato plants, fruits developed without pollination and therefore were seedless [27]. Fruit size and morphology of the seedless fruits were comparable to those of seeded fruits of the parental line. There are no examples in the literature of parthenocarpy obtained by alteration of auxin transport, although the treatment of flowers with inhibitor of auxin transport induces fruit growth in the absence of pollination. Seedless fruits have been obtained by down regulating Chalcone synthase (*Chs*), the first gene in the flavonoid biosynthetic pathway [28]. Interestingly, loss of CHS activity in *A. thaliana* caused an increase in polar auxin transport. It is possible that in *Chs*-silenced tomato, parthenocarpy results from an altered distribution of auxin caused by the reduced level of flavonoids.

## 3. Gibberellins

Gibberellins (GAs) are a second group of phytohormones that plays a prominent role in coordinating fruit growth and seed development. Active gibberellins (GA1 or GA3) are able to induce fruit set in several horticultural species and in the model species *A. thaliana* [1,13]. However, tomato gibberellin-induced fruits are smaller than seeded fruits suggesting that other signals are required for tomato fruit growth and development [29]. Increased levels of gibberellin have been detected in pollinated ovary together with an increased expression of GA biosynthetic genes [30]. On the other hand, the application of inhibitors of GA biosynthesis limits fruit growth [31]. The role of gibberellins in fruit set was also supported by the analysis of the parthenocarpic tomato mutans *pat*, *pat2* and *pat3/4*. These mutants show increased level of GAs and an enhanced expression of GA biosynthetic genes [32,33]. 

A synergistic effect of auxin and gibberellin on fruit growth has been observed in pea and tomato suggesting that the two phytohormones interact in regulating fruit development [31,34]. Auxin and gibberellin may act in parallel or in a sequential way on fruit set. Data recently obtained in *A. thaliana* and in tomato suggest a hieralchical scheme of interaction where GA acts downstream of auxin [13,31]. In tomato, auxin-induced fruit initiation is mediated by GAs and in ovaries treated with auxins GA biosynthetic genes are induced [31]. Similar results were obtained in *A. thaliana* where fertilization triggers an increase in auxin response in the ovules and the activation of GA synthesis [13]. Therefore the molecular mechanisms by which hormones regulate fruit set seem very similar in fleshy (*i.e.*, tomato) and dry fruit (*i.e.*, *A. thaliana*) implicating auxin-induced activation of gibberellin synthesis and signaling.

Seedless fruits have been produced by genetically alterating GA signaling (Table 1). The depletion of SlDELLA proteins which are negative regulators of GA signal transduction pathway, allowed to overcome the growth arrest normally imposed on the ovary at anthesis [35]. Antisense *SlDELLA*-engineered tomato fruits were seedless, but smaller in size and elongated in shape compared with pollinated fruits. Cell number estimations showed that fruit set, resulting from reduced *SlDELLA* expression, arose from activated cell elongation at the longitudinal and lateral axes of the fruit pericarp. Interestingly also the quadruple-DELLA loss-of-function mutant of *A. thaliana* displays the parthenocarpic phenotype [13].

## 4. Ethylene

In unpollinated tomato ovaries ethylene biosynthesis and signaling genes are highly expressed [7] and it is known that in many plant species pollination itself triggers a transient increase in ethylene production in pistils. After fruit initiation ethylene biosynthesis starts to decline [7,8]. The production of pollination–induced ethylene is thought to play a role in coordinating ovary growth and flower senescence, events that are associated to fruit set. Transcriptome analysis of tomato ovaries has revealed shifts in a high number of ethylene related genes in the transition from flower to fruits [7,8,9]. These genes comprise both ethylene biosynthesis genes and ethylene signal transduction pathway genes. It was suggested that the down regulation of ethylene biosynthesis after fruit initiation is related to the release of the dormant state of the ovary [7]. In this respect, the function of ethylene might be antagonistic to that of auxin and gibberellin in that it would prevent ovary growth before pollination. On the other hand, it was observed that enhanced ethylene response leads to parthenocarpic fruit development (Table 1). 

**Table 1:** Seedless fruit production by genetically engineering hormone-related genes. Sl = *Solanum lycopersicum*; At= *Arabidopsis thaliana.*

Gene	Function	Genetic modification	Fruit Phenotype	Vegetative alterations	Reference
*DeH9-iaaM*	Auxin synthesis	Ovule specific transgene expression	Obligate and facultative parthenocarpy, seedless, early fruit growth, normal size and shape. (tobacco, eggplant, tomato, raspberry, cucumber)	no	[17,18,19,20]
*rolB*	Auxin response	Ovary/ fruit specific transgene expression	Obligate and facultative parthenocarpy, seedless, size and shape similar to wt. (tomato)	no	[27]
*SlIAA9*	Auxin signaling	Antisense downregulation, constitutive promoter	Parthenocarpy, seedless, early fruit growth, normal size and shape. (tomato)	yes	[22]
*AtARF8*	Auxin signaling	Expression of mutant AtARF8-4 gene	Parthenocarpy, seedless/pseudoembryos, size and shape similar to wt. (tomato, A. thaliana)	not indicated	[24]
*SlARF7*	Auxin signaling	RNAi-mediated silencing, constitutive promoter	Parthenocarpy, seedless, altered shape. (tomato)	no	[25]
*SlAUCSIA*	Auxin response	RNAi-mediated silencing, phloem-specific promoter	Facultative and obligate parthenocarpy, seedless, reduced size. (tomato)	yes	[26]
*SlChs*	Flavonoid biosynthesis (auxin transport?)	RNAi-mediated silencing, constitutive promoter	Parthenocarpy, seedless, altered size, shape and colour. (tomato)	no	[28]
*SlDELLA*	Gibberellin signaling	Antisense downregulation, constitutive promoter	Facultative parthenocarpy, seedless, reduced size, altered morphology. (tomato)	yes	[35]
*SlTPR1*	Ethylene signaling	Overexpression	Parthenocarpy, seedless, altered morphology. (tomato)	yes	[36]

Lin *et al.* [36] have identified in tomato a new component of the ethylene signalling, SlTPR1 which binds ethylene receptors NR and LeETR1. Overexpression of SlTPR1 induced parthenocarpic fruit development and many other developmental alterations related to ethylene and auxin [36]. Interestingly, 35S::SlTPR1 tomato plants showed reduced expression of the auxin response gene *SlIAA9*. *SlIAA9* expression is known to be inhibited by ethylene and antisense silencing of *SlIAA9* results in early and pollination-independent fruit development [22]. This finding suggests that the hormonal regulation of fruit initiation might involve cross talk between ethylene and auxin.

## 5. Other hormones

Cytokinins and brassinosteroids are other types of phytohormones thought to be involved in fruit growth promotion [1,37]. The application of cytokinins to flowers before fertilization triggers in some species (*i.e.*, pea) the onset of fruit growth. Furthermore, cytokinins as well as auxins and GAs are accumulated after fertilization [1]. As cytokinins regulate cell division, it is plausible to associate this hormone with the first phase of fruit growth which is characterized by a marked increase in cells number. Indeed, a correlation between cytokinins levels and cell division activities has been observed in tomato fruits [38]. Elevated concentrations of cytokinins are detected in developing seeds, suggesting that cytokinins play a role in synchronizing embryo/seed development and fruit growth. 

At a molecular level, cytokinins related genes did not appear to be strongly controlled at a transcriptional level during fruit set and no differences in cytokinins related gene expression were found between parthenocarpic and non parthenocarpic tomato fruits [7,8].Thus the role of this hormone is still elusive and production of seedless fruit by genetic alteration of cytokinins synthesis or action has never been reported.

Brassinosteroids (BRs) are steroid hormones that play a prominent role in many plant developmental processes. BRs have promoting effects on plant growth and interact with auxins in a synergistic way. The effects of these hormones on early fruit growth were addressed in a recent work that describes BR activity in cucumber [39]. The application of BR induced parthenocarpic growth in cucumber accompanied by active cell division, whereas the application of an inihibitor of BR biosynthesis blocked fruit growth in a parthenocarpic cultivar [39]. It was suggested that the promoting effect on cucumber fruit growth is linked to the induction of cell cycle-related genes. Interestingly, application of BR to unpollinated cucumber flowers produced seedless fruits similar to those of the pollinated flowers. In tomato, however, brassinosteroids are apparently not involved in fruit set and growth because the Micro-Tom cultivar bearing a mutation in brassinosteroid biosynthesis gene does not display abnormalities in fruit development [40,41]. Furthermore, brassinolide application to tomato ovaries does not induce parthenocarpic fruit set [41].

## 6. Conclusions

In the last years molecular analyses of pollinated-dependent and pollinated -independent fruit set have revealed a complex hormonal regulatory network. The role of phytohormones as well as their cross-talk is of fundamental importance in fruit set regulation.

Quite unexpectedly, genetic engineering has demonstrated that it is possible to uncouple fruit growth from fertilization and obtain seedlessness in horticultural plants by modification of single genes [42]. We can speculate that despite the molecular complexity of the process, there is a hierarchical series of events or many parallel processes controlled by single genes. Basing on the current knowledge, the hypothesis of a hierarchical mode of regulation where auxin plays the prominent role is the most plausible [13,14].

A genetic method for obtaining seedless fruits is valuable for the market only if fruit quality and productivity are not curtailed. Furthermore the horticultural plants should not display any vegetative or reproductive alterations except for the lack of seeds. To reach this goal it is important to consider the strength and the spatial and temporal expression of the promoter. For restricting the effect of the genetic manipulation to the fruit it is crucial to choose a promoter that drives gene expression specifically or prevalently in the ovules, ovaries or other fruit tissues. Furthermore, in same cases it could be convenient to express the transgene only at a specific stage of fruit development (e.g., fruit set), whereas in other cases the transgene should be expressed during the entire period of fruit growth. The latter is the case of auxin biosynthecic genes. *DefH9-iaaM* gene is expressed in the ovules and placenta but also in the tissues derived from them, allowing the synthesis of auxin also in later stages of fruit growth. This continuous supply of auxin produces seedless fruits that are equal or bigger in size compared to pollinated fruits. The strength of expression is also important because hormones normally function at low concentrations and in narrow intervals; too low or too high level of phytohormones, as well as drastic change in hormone sensitivity, can lead to phenotypic anormalities. Although still incomplete, our knowledge of the hormonal regulation of fruit set could be used to develop new genetic methods for the production of marketable seedless plants based on the modification of endogenous genes (cis-genesis) rather than on the use of exogenous DNA sequences (trans-genesis). Based on the present knowledge, the best targets for genetic manipulation of fruit set can be envisaged in the components of auxin signalling pathway. In this respect, both transgenic and non-transgenic approaches such as TILLING could have promising applications for several reasons: activation of auxin pathway appears to be the first post-pollination event in a hierarchic scheme of signalling and there is a deal of information on auxin response genes, auxin transport and metabolism. In addition, it seems feasible to obtain auxin-based seedless plants without alteration of vegetative growth and fruit quality.
